# Patterns of nicotine dependence in four Eastern European countries

**DOI:** 10.1186/s12889-015-2537-0

**Published:** 2015-11-28

**Authors:** Dorota Kaleta, Kinga Polańska, Przemysław Korytkowski, Bukola Usidame, Leokadia Bąk-Romaniszyn

**Affiliations:** Department of Tobacco Control, Preventive Medicine Department, Medical University of Lodz, 90-752 Lodz, Poland; Faculty of Computer Science and Information Technology, West Pomeranian University of Technology in Szczecin, 71-210 Szczecin, Poland; Department of Public Policy, University of Massachusetts, Boston, USA; Department of Nutrition in Digestive Tract Diseases, Medical University of Lodz, 93-338 Lodz, Poland

**Keywords:** Smoking, Tobacco, Nicotine dependence, Adults, GATS

## Abstract

**Background:**

Evidence of patterns of nicotine dependence, although crucial for developing and implementing effective tobacco control strategies, is limited in the Eastern European countries. The purpose of this study was to evaluate the correlates of high nicotine dependence among adults in Poland, Romania, the Russian Federation and Ukraine.

**Methods:**

The data used in the current analysis is available from the Global Adult Tobacco Survey (2009–2011). Nicotine dependence was assessed using the Heaviness of Smoking Index (HSI), which covers two measures: reported cigarettes smoked per day and time to the first cigarette upon waking. Based on a six-point scale of HSI, nicotine dependence was categorized into low to moderate (score 0–3), and high dependence (score 4–6). Out of 31,936 completed interviews, we used data from 8229 daily smokers.

**Results:**

The study results indicate that more than 25 % of daily smokers were highly dependent on nicotine. Higher odds of high nicotine dependence were identified for males (OR = 1.5 in Poland and Romania, OR = 2.7 in Russia; *p* ≤ 0.001), people between 50–59 years of age (the highest odds in Romania; OR = 4.8; *p* ≤ 0.001) and those who had started smoking at a young age (the highest odds in Romania, OR = 5.0; *p* ≤ 0.001). Having fewer restrictions on smoking at home was significantly associated with a high level of nicotine dependence (the highest odds in Romania, OR = 3.0; *p* ≤ 0.001). A high proportion of the participants had no interest in quitting smoking, with a statistically significantly higher percentage observed among smokers highly dependent on nicotine compared to the less addicted (*p* ≤ 0.01).

**Conclusions:**

Smokers highly dependent on nicotine constitute a quarter of the Romanian group of daily smokers and even more in the remaining three analyzed countries. Similar patterns of nicotine dependence were observed in all of the investigated countries showing that male gender, younger age at the smoking onset, and fewer restrictions on smoking at home were significantly associated with higher nicotine dependence. The study highlighted the fact that a high proportion of the participants had no interest in quitting smoking. These results underscore importance of policy measures as well as prevention and cessation interventions for smokers who are highly dependent on nicotine, which need to take into account the social gradient in smoking patterns.

## Background

According to the World Health Organization (WHO), tobacco smoking kills more Europeans than any other preventable risk factor [1]. Compared to other regions, the WHO European Region has one of the highest rates of smoking, and the highest proportion of deaths attributable to tobacco. In addition to the loss to society caused by premature deaths, tobacco consumption also leads to higher health care costs and decreased economic productivity due to more cases of absenteeism from work, premature retirements or deaths [2, 3].

The four Eastern European countries, namely Poland, Romania, the Russian Federation and Ukraine, are among the countries experiencing the highest smoking rates and consequently highest tobacco-related health burden. Smoking prevalence among Russian and Ukrainian men is one of the highest in the world with more than, respectively, 60 % and 50 % of men consuming tobacco [4, 5]. It needs to be pointed out that male life expectancy in Russia dropped from 64 years in 1989 to 59 years in 2008, and from 66.2 years in 1989 to 62.2 years in 2007 in Ukraine, mainly due to tobacco consumption [6]. Smoking prevalence for women, though lower than for men, is still 22 % in Russia and 11 % in Ukraine [4, 5]. In Poland and Romania 37 % of adult men reported tobacco smoking, either regular or occasional [7, 8]. For women, these percentages are 21 % and 17 %, respectively [7, 8].

As a result of public health and regulatory activities, an overall reduction in smoking prevalence in Europe has been observed in recent years. Despite this, the Eastern European countries lag behind the West in implementing tobacco control measures. In addition, the main decrease in tobacco consumption has been achieved in the middle and high-income groups, causing a significant widening of inequalities [9]. WHO suggests that national population-based tobacco control policies are important, but are unlikely to significantly reduce inequalities without additional measures. When developing tobacco control policies at European, national and local levels, it is essential to consider equity implications with the best available evidence [10]. According to the WHO recommendations, understanding methods that reduce tobacco use across all social groups is critical for addressing overall tobacco consumption [11–14].

Apart from monitoring prevalence of smoking, understanding nicotine dependence and its determinants are also crucial for developing and implementing effective tobacco control strategies [15–17]. The patterns of nicotine dependence in the Eastern European countries are not well documented. This is a clear barrier for the effective tobacco control initiatives [5].

Therefore, we explored factors associated with nicotine dependence in four Eastern European countries.

## Methods

### Study design and population

The data used in the current analysis is available from the Global Adult Tobacco Survey (GATS) [18–20], a global standard used to systematically monitor adult tobacco use and track key tobacco control indicators. Country-specific, anonymous GATS data was freely available from the Centers for Disease Control and Prevention (CDC) Global Tobacco Surveillance System Data website. The target population included all non-institutionalized men and women aged 15 years or older who considered a given country to be their usual place of residence. The survey methodology has been previously described in detail [14, 18–20]. GATS meets standard protocol measures that allow country-by-country comparison on a global scale. The survey has been seen as a part of the Bloomberg Global Initiative to Reduce Tobacco Use by the Bloomberg Philanthropies, who support the survey in a bid to fill the gap for measuring adult tobacco use globally. The GATS was conducted in Poland, Romania, the Russian Federation and Ukraine by the National Implementing Agencies under the supervision and approval of the Ministries of Health and the World Health Organization Country Offices. All questionnaire contents were reviewed and approved by the Questionnaire Expert Review Committees. In all countries, the Ministries of Health revised and approved the study protocols and also appointed two committees – the GATS Scientific Committee and the GATS Steering Committee – who handled the scientific, ethical and technical coordination of the study. The WHO Country Offices and the CDC also participated. The study obtained informed consent from all participants to participate in the study. Informed consent was obtained from parents or guardian in the case of participants under the age of 16. Accordingly, with the GATS requirements and procedures, a geographically clustered, multistage sampling methodology was used to identify the specific households that Field Interviewers contacted. First, each country was divided into Primary Sampling Units, then segments within the Primary Sampling Units, and households within the segments, from which a random sample of households was selected to participate in GATS. The GATS interview was composed of a Household Questionnaire and an Individual Questionnaire. These questionnaires were utilized during face to face interviews and recorded on an electronic data collection device. Out of 31,936 completed interviews we utilized self-reported data on smoking from 8229 daily smokers (person who smokes at least one cigarette a day) aged 26 and above from the four mentioned earlier Eastern European countries.

The overall survey response rates were, as follows: Poland 65.1 %, Romania 88.5 %, Russia 97.7 % and Ukraine 76.2 %.

### Study variables

The outcome variable was the level of nicotine dependence assessed by the Heaviness of Smoking Index (HSI) [21–25]. HSI covers two measures, which are identified as the most predictive for nicotine dependence: reported cigarettes smoked per day (How many cigarettes do you typically smoke per day?) and time to the first cigarette upon waking (On the days that you smoke, how soon after you wake up do you have your first cigarette?). Based on a six-point scale calculated from these two measures: 1) the number of cigarettes smoked per day (10 or fewer: 0 points, 11–20: 1 point, 21–30: 2 points, 31 or more cigarettes: 3 points) and 2) the time to the first cigarette after waking (within 5 min: 3 points, 6–30 min: 2 points, 31–60 min: 1 point, after 60 min: 0 points), nicotine dependence was categorized into either low to moderate (0–3) or high (4–6). The HSI has been revealed to be a reliable and a valid measure of the severity of nicotine dependence, associated with objective measures such as cotinine levels or alveolar carbon monoxide [21–25]. The HSI has been also found to identify a similar dependent population to the Fagerstrom Tolerance Questionnaire (FTQ) and the Fagerstrom Test for Nicotine Dependence (FTND) [25].

The variables applied to determine nicotine dependence associations were gender (male, female) and age. The age of the respondents was categorized into five groups: 26–29, 30-39, 40–49, 50–59, and ≥60 years old. The age at smoking onset – the age at which the respondents began to smoke tobacco on a regular basis – was also taken into account (<14, 14–17, 18–20, 21 years or over). Furthermore, the socio-economic status (including education, economic activity, place of residence and Asset Index (AI)) was assessed. Educational attainment was regarded as completed: primary, vocational, secondary or high education. Economic activity differentiated the subjects who were not economically active (pupils, students, homemakers, retirees, and pensioners due to disability), the currently employed, and the unemployed. The place of residence differentiated rural from urban settings. The Asset Index reflected ownership of different household items and was calculated based on a cumulative score of possession of the following assets: functioning electricity, flushing toilet, fixed telephone, mobile telephone, television, radio, refrigerator, car, washing machine, computer and internet access. The score was categorized into high, medium, low. Rules regarding smoking at home were categorized, as follows: smoking is allowed everywhere, smoking is prohibited with some exceptions from the rules, smoking is prohibited in all areas, and no rules. Awareness of smoking health consequences was sought using the question: Do you think that tobacco smoking causes serious diseases? The respondents were classified as being aware if they answered “yes”, and unaware if they answered “no” or “do not know”. Likewise, we assessed awareness of health consequences of exposure to environmental tobacco smoke (ETS). In addition, we evaluated support for tobacco control policies among the respondents by distinguishing between high, medium and low levels of support. This measure was based on 8 questions specifying different tobacco control policy items. The cumulative score was divided into: supporting 4–5 policies (high level), 2–3 policies (medium level) and 0–1 policy (low level). Finally, we also focused on any intentions to quit, attempts at quitting during the past 12 months and healthcare provider encouragement or advice to quit smoking. The smokers were asked whether they had visited a healthcare professional in the year prior to the survey and if they answered “yes” whether they have been asked about their smoking habits and whether they had received quitting advice from the healthcare professional.

### Statistical analysis

The STATISTICA Windows XP version 10.0 program was used to carry out the statistical analysis. Initially, a descriptive analysis for all the variables involved in the analysis was completed. Categorical variables were studied by the use of the chi-square test. Univariate and multivariable logistic regression analyses with results being presented as odds ratios (OR) with 95 % confidence intervals were applied to the study associates of high nicotine dependence among adults in the four studied countries. In the multivariable analyses, all statistically significant characteristics (*p* < 0.05) were simultaneously included. To test multicollinearity between the variables, the variance inflation factor (VIF) was calculated. VIFs in all except one (VIF = 5.1 for people older than 60 years) were lower than 4, which indicates that the assumption of reasonable independence among predictor variables was met.

## Results

### Characteristics of the study population

The characteristics of the sample are described in Table 1. Women represented about 17 % of the studied daily smokers aged 26 years and older in Russia, 31 % in Romania, 41 % in Poland and 50 % in Ukraine. In all the countries, the lowest proportion of participants was noted in the youngest (26–29 years of age) and oldest (≥60 years of age) age categories. The highest proportion of the study subjects that were daily smokers with higher education was noted in Russia and the lowest in Poland. In all the countries more than 60 % of the subjects included in the analysis were employed. Urban areas as the place of residence were indicated by slightly more than 50 % of the study participants. A high AI category was noticed for 78 % of the daily smokers from Poland and only for 39 % from Russia. About 60 % of the study subjects in Poland and Romania indicated that smoking was allowed at home or that there were no rules regarding smoking at home. Such categories were indicated by fewer Ukrainians and Russians. In all the analyzed countries the proportions of people who were aware of health consequences of ETS exposure were slightly lower compared to the proportion of the people who were aware of health consequences of active smoking. A high level of support for tobacco control was specified by around 50 % of the study sample in all the countries.

**Table 1:** High nicotine dependence by selected socio-demographic characteristics in men aged 26 years and over – univariable logistic regression

Variable	Poland (*N* = 1939)				Romania (*N* = 867)				Russia (*N* = 3558)				Ukraine (*N* = 1865)			
	N	n	%	OR (95 % CI)	N	n	%	OR (95 % CI)	N	n	%	OR (95 % CI)	N	n	%	OR (95 % CI)
Sex	*N* = 1939				*N* = 867				*N* = 3558				*N* = 1865			
Female	785	170	21.7	1.0 (Ref.)	272	50	18.4	1.0 (Ref.)	600	80	13.3	1.0 (Ref.)	240	25	10.4	1.0 (Ref.)
Male	1154	364	31.5	1.7 (1.4–2.1)^a^	595	170	28.6	1.8 (1.3–2.5)^b^	2958	969	32.8	3.2 (2.5–4.1)^a^	1625	458	28.2	3.4 (2.2–5.2)^a^
Age (years)	*N* = 1939				*N* = 867				*N* = 3558				*N* = 1865			
26–29	182	33	18.1	1.0 (Ref.)	88	14	15.9	1.0 (Ref.)	399	96	24.1	1.0 (Ref.)	217	27	12.4	1.0 (Ref.)
30–39	453	103	22.7	1.4 (0.9–2.1)	207	37	17.9	1.2 (0.7–2.1)	945	225	23.8	0.99 (0.8–1.3)	541	136	25.1	2.4 (1.5–3.7)^a^
40–49	523	154	29.4	1.9 (1.2–2.9)^b^	233	75	32.2	2.8 (1.7–4.6)^a^	886	256	28.9	1.3 (0.98–1.7)	433	125	28.9	2.9 (1.8–4.5)^a^
50–59	521	174	33.4	2.3 (1.5–3.4)^a^	209	67	32.1	2.7 (1.6–4.4)^a^	845	304	36.0	1.8 (1.4–2.3)^a^	385	116	30.1	3.0 (1.9–4.8)^a^
≥60	260	70	26.9	1.7 (1.04–2.7)^c^	130	27	20.8	1.5 (0.8–2.7)	483	168	34.8	1.7 (1.3–2.3)^a^	289	79	27.3	2.7 (1.6–4.3)^a^
Age at smoking onset	*N* = 1880				*N* = 867				*N* = 3557				*N* = 1840			
<14	44	20	45.5	3.4 (1.8–6.3)^a^	52	21	40.4	3.6 (1.9–6.7)^a^	288	151	52.4	3.9 (2.9–5.3)^a^	175	78	44.6	3.7 (2.5–5.6)^a^
14–17	583	220	37.7	2.4 (1.8–3.3)^a^	266	98	36.8	2.8 (1.8–4.4)^a^	1291	420	32.5	1.7 (1.4–2.1)^a^	634	198	31.2	2.4 (1.7–3.3)^a^
18–20	806	186	23.1	1.2 (0.9–1.6)	338	69	20.4	1.5 (0.95–2.3)	1229	313	25.5	1.2 (0.98–1.5)	678	147	21.7	1.5 (1.04–2.0)^c^
≥21	447	89	19.9	1.0 (Ref.)	211	32	15.2	1.0 (Ref.)	749	164	21.9	1.0 (Ref.)	353	57	16.1	1.0 (Ref.)
Education	*N* = 1934				*N* = 860				*N* = 3556				*N* = 1861			
Primary	294	101	34.4	2.6 (1.7–4.1)^a^	58	19	32.8	2.3 (1.2–4.5)^a^	91	41	45.1	3.0 (1.9–4.7)^a^	122	48	39.3	4.4 (2.7–7.3)^a^
Vocational	746	229	30.7	2.2 (1.5–3.3)^a^	200	53	26.5	1.7 (0.98–2.9)	1349	406	30.1	1.6 (1.3–1.93)^a^	802	181	22.6	2.0 (1.4–2.9)^a^
Secondary	696	170	24.4	1.6 (1.1– 2.4)^a^	476	127	26.7	1.6 (0.97–2.5)	1228	412	33.6	1.9 (1.5– 2.3)^a^	649	215	33.1	3.4 (2.3–4.9)^a^
High	198	33	16.7	1.0 (Ref.)	126	22	17.5	1.0 (Ref.)	888	190	21.4	1.0 (Ref.)	288	37	12.8	1.0 (Ref.)
Occupational classification	*N* = 1939				*N* = 863				*N* = 3554				*N* = 1860			
Economically not active	587	168	28.6	1.1 (0.92– 1.4)	181	40	22.1	0.9 (0.7-1.3)	590	194	32.9	1.3 (1.1– 1.6)^c^	357	98	27.5	1.3 (1. 1–7)^c^
Employed	1187	307	25.9	1.0 (Ref.)	561	136	24.2	1.0 (Ref.)	2611	720	27.6	1.0 (Ref.)	1185	265	22.4	1.0 (Ref.)
Unemployed	165	59	35.8	1.6 (1.1–2.2)^b^	121	43	35.5	1.8 (1.2–2.8)^b^	353	134	38.0	1.6 (1.2–2.1)^a^	318	120	37.7	2.2 (1.6–2.8)^a^
Place of residence	*N* = 1939				*N* = 867				*N* = 3558				*N* = 1865			
Rural	911	255	28.0	1.04 (0.9–1.3)	355	99	27.9	1.3 (0.96–1.7)	1676	555	33.1	1.4 (1.2–1.6)^a^	906	274	30.2	1.6 (1.3–1.9)^a^
Urban	1028	279	27.1	1.0 (Ref.)	512	121	23.6	1.0 (Ref.)	1882	494	26.2	1.0 (Ref.)	959	209	21.8	1.0 (Ref.)
Asset Index	*N* = 1922				*N* = 856				*N* = 3558				*N* = 1865			
High	1495	390	26.1	1.0 (Ref.)	447	108	24.2	1.0 (Ref.)	1399	360	25.7	1.0 (Ref.)	755	160	21.2	1.0 (Ref.)
Middle	377	116	30.8	1.3 (0.98–1.6)	293	78	26.6	1.1 (0.8–1.6)	1956	605	30.9	1.3 (1.1–1.5)^b^	884	243	27.5	1.4 (1.1–1.8)^b^
Low	50	24	48.0	2.6 (1.5–4.6)^a^	116	36	31.0	1.6 (1.03–2.4)^c^	203	84	41.4	2.0 (1.5–2.8)^a^	226	80	35.4	2.0 (1.5–2.8)^a^
Rules regarding smoking at home	*N* = 1934				*N* = 860				*N* = 3553				*N* = 1864			
Smoking is allowed	975	313	32.1	2.5 (1.8–3.6) ^a^	446	138	30.9	1.9 (1.3–2.8)^a^	945	333	35.2	1.7 (1.4–2.1)^a^	344	119	34.6	2.1 (1.6–2.7)^a^
Smoking is prohibited– with some exceptions	488	114	23.4	1.6 (1.1–2.4)^c^	162	26	16.0	0.9 (0.6–1.5)	1296	377	29.1	1.3 (1.1–1.6)^b^	604	169	28.0	1.5 (1.2–1.9)^a^
Smoking is completely prohibited	270	43	15.9	1.0 (Ref.)	220	41	18.6	1.0 (Ref.)	1059	254	24.0	1.0 (Ref.)	847	173	20.4	1.0 (Ref.)
No rules.	201	62	30.8	2.4 (1.5–3.7)^a^	32	15	46.9	4.4 (2.1–9.2)^a^	253	83	32.8	1.6 (1.2–2.1)^b^	69	22	31.9	1.8 (1.1–3.1)^c^
Awareness of smoking health consequences	*N* = 1782				*N* = 861				*N* = 3417				*N* = 1753			
Yes	1566	399	25.5	1.0 (Ref)	812	202	24.9	1.0 (Ref.)	3013	883	29.3	1.0 (Ref.)	1596	394	24.7	1.0 (Ref.)
No	216	76	35.2	1.6 (1.2–2.2)^b^	49	17	34.7	1.7 (0.9–3.0)	404	117	29.0	0.98 (0.8–1.2)	157	55	35.0	1.5 (1.1–2.0)^b^
Awareness of ETS health consequences	*N* = 1624				*N* = 854				*N* = 3218				*N* = 1604			
Yes	1312	353	26.9	1.0 (Ref.)	784	188	24.0	1.0 (Ref.)	2544	722	28.4	1.0 (Ref.)	1402	332	23.7	1.0 (Ref.)
No	312	91	29.2	1.1 (0.9–1.5)	70	25	35.7	1.9 (1.2–3.1)^b^	674	215	31.9	1.2 (0.98–1.4)	202	74	36.6	1.6 (1.2–2.0)^a^
Level of support for tobacco control	*N* = 1476				*N* = 839				*N* = 3558				*N* = 1865			
High	829	202	24.4	1.0 (Ref.)	353	82	23.2	1.0 (Ref.)	1950	532	27.3	1.0 (Ref.)	936	220	23.5	1.0 (Ref.)
Medium	416	137	32.9	1.5 (1.2–2.0)^a^	431	111	25.8	1.1 (0.8–1.5)	1314	403	30.7	1.2 (1.01–1.4)^c^	881	256	29.1	1.3 (1.1.–1.6)^c^
Low	231	64	27.7	1.2 (0.9–1.7)	55	19	34.5	1.53 (0.9–2.7)	294	114	38.8	1.7 (1.3–2.2)^a^	48	7	14.6	0.6 (0.3–1.3)

^a^ *p* ≤ 0.001

^b^ *p* ≤ 0.01

^c^ *p* ≤ 0.05

N- total number of daily smokers

n - daily smokers highly dependent on nicotine (Heaviness of Smoking Index: score 4–6)

OR- odds ratio for high nicotine dependence by selected characteristics

95 % CI-95 % confidence interval

Ref. – reference group

### Patterns of nicotine dependence

Based on the HSI about 30 % of Russians, 28 % of Poles, 26 % of Ukrainians and 25 % of Romanians were categorized as highly dependent on nicotine (Table 1). The univariate logistic regression analysis of the factors influencing the level of nicotine dependence is presented in Table 1, and the multivariable analysis in Table 2. Generally, the results of the multivariable model are in agreement with that obtained in the univariate analysis. Greater odds of being highly dependent on nicotine were identified for males (with OR = 1.5; *p* ≤ 0.001 in Poland and Romania, to OR = 2.7; *p* ≤ 0.001 in Russia). The significantly greater odds of high dependence were noted in all the analyzed countries among people 50–59 years of age, compared to the youngest age group (with OR = 1.7; *p* ≤ 0.001 in Russia, to OR = 4.8; *p* ≤ 0.001 in Romania). A younger age at smoking onset (<17 years of age) was a significant predictor of high nicotine dependence in all the analyzed countries. The results relating to the impact of educational level on high nicotine dependence are not consistent across all the analyzed countries (the increased risk was observed among the people with primary and vocational levels of education in Poland, primary school in Ukraine and among those with secondary level of education in Russia and Ukraine). Among the analyzed countries only in Ukraine the unemployed people had significantly higher odds of being heavy dependent on nicotine compared to the employed people (OR = 1.6; *p* ≤ 0.01). In Russia a slightly increased risk of being highly nicotine dependent was noted among rural populations compared to those living in urban areas (OR = 1.2; *p* ≤ 0.05). The likelihood of higher nicotine dependence was significantly higher for the people who had indicated that smoking was allowed at home or for those who indicated no rules regarding smoking at home, compared to the populations indicating a total ban on smoking in the home environment; with the highest odds for no rules (OR = 3.0 *p* ≤ 0.001 in Romania). Awareness of health consequences of active and passive smoking as well as economic status based on the Asset Index did not have any significant impact on the level of nicotine dependence in any of the analyzed countries (*p* > 0.05). Medium level of support for tobacco control in the two countries, namely Poland and Russia, was a significant risk factor for HSI higher than 3.

**Table 2:** High nicotine dependence by selected socio-demographic characteristics in men aged 26 years and over – Multivariable logistic regression^a^

Variable	Poland	Romania	Russia	Ukraine
	OR (95 % CI)	OR (95 % CI)	OR (95 % CI)	OR (95 % CI)
Sex				
Female	1.0 (Ref.)	1.0 (Ref.)	1.0 (Ref.)	1.0 (Ref.)
Male	1.5 (1.1–2.0)^b^	1.5 (1.01–2.2)^d^	2.7 (2.1–3.5)^b^	2.6 (1.7–4.2)^b^
Age (years)				
26–29	1.0 (Ref.)	1.0 (Ref.)	1.0 (Ref.)	1.0 (Ref.)
30–39	1.4 (0.8–2.3)	1.5 (0.9–2.8)	1.0 (0.8–1.3)	2.4 (1.5–3.9)^b^
40–49	1.8 (1.1–3.1)^d^	4.1 (2.3–7.1)^b^	1.3 (1.0–1.7)	2.8 (1.8– 4.6)^b^
50–59	1.9 (1.2–3.3)^c^	4.8 (2.7–8.4)^b^	1.7 (1.3–2.3)^b^	2.9 (1.8–4.8)^b^
≥60	1.5 (0.8–2.9)	2.0 (1.0–4.1)^d^	1.4 (0.9–2.0)	2.2 (1.1–4.3)^d^
Age at smoking onset				
<14	3.8 (1.8–8.4)^b^	5.0 (2.4–10.2)^b^	3.0 (2.2–4.1)^b^	3.1 (2.0–4.9)^b^
14–17	2.5 (1.7–3.6)^b^	3.5 (2.1–5.8)^b^	1.5 (1.2–1.9)^b^	1.9 (1.3–2.7)^b^
18–20	1.2 (0.8–1.6)	1.7 (1.0–2.7)^c^	1.1 (0.9–1.4)	1.2 (0.8–1.7)
≥21	1.0 (Ref.)	1.0 (Ref.)	1.0 (Ref.)	1.0 (Ref.)
Education				
Primary	2.6 (1.4–4.9)^c^	1.2 (0.5–2.9)	1.6 (0.9–2.6)	3.0 (1.7–5.4)^b^
Vocational	2.0 (1.2–3.6)^c^	1.2 (0.6–2.1)	1.2 (1.0–1.5)	1.5 (1.0–2.2)
Secondary	1.7 (0.95–3.0)	1.2 (0.7–2.0)	1.3 (1.0–1.6)^d^	2.3 (1.5– 3.5)^b^
High	1.0 (Ref.)	1.0 (Ref.)	1.0 (Ref.)	1.0 (Ref.)
Occupational classification				
Economically not active	1.1 (0.8–1.6)	0.9 (0.6–1.4)	1.0 (0.7–1.4)	0.9 (0.6–1.5)
Employed	1.0 (Ref.)	1.0 (Ref.)	1.0 (Ref.)	1.0 (Ref.)
Unemployed	1.4 (0.9–2.1)	1.5 (0.9–2.3)	1.2 (0.9–1.6)	1.6 (1.1–2.1)^c^
Place of residence				
Rural			1.2 (1.0–1.4)^d^	1.3 (1.0–1.6)
Urban			1.0 (Ref.)	1.0 (Ref.)
Asset Index				
High	1.0 (Ref.)	1.0 (Ref.)	1.0 (Ref.)	1.0 (Ref.)
Middle	1.1 (0.8–1.5)	0.8 (0.5–1.1)	1.3 (0.9–1.8)	0.8 (0.6–1.2)
Low	0.9 (0.4–1.8)	0.7 (0.4–1.3)	1.2 (1.0–1.4)	0.8 (0.6–1.0)
Rules regarding smoking at home				
Smoking is allowed	1.9 (1.2–3.0)^c^	2.0 (1.3–3.0)^b^	1.8 (1.4–2.2)^b^	2.1 (1.5–2.9)^b^
Smoking is prohibited– with some exceptions	1.7 (1.1–2.6)^d^	1.0 (0.6–1.7)	1.3 (1.1–1.6)^c^	1.6 (1.2–2.1)^b^
Smoking is completely prohibited	1.0 (Ref.)	1.0 (Ref.)	1.0 (Ref.)	1.0 (Ref.)
No rules	2.0 (1.2–3.5)^c^	3.0 (1.4–6.8)^b^	1.5 (1.1–2.1)^d^	1.4 (0.8–2.5)
Awareness of smoking health consequences				
Yes	1.0 (Ref.)			1.0 (Ref.)
No	1.3 (0.9–2.0)			1.0 (0.7–1.5)
Awareness of ETS health consequences				
Yes		1.0 (Ref.)		1.0 (Ref.)
No		1.6 (0.9–2.7)		1.4 (0.9–2.0)
Level of support for tobacco control				
High	1.0 (Ref.)		1.0 (Ref.)	1.0 (Ref.)
Medium	1.6 (1.1–2.1)^c^		1.4 (1.1–1.7)^c^	1.2 (0.9–1.6)
Low	0.9 (0.6–1.3)		1.3 (0.7–2.2)	0.9 (0.3–2.9)

^a^Fully adjusted model including stat

^b^ *p* ≤ 0.001

^c^ *p* ≤ 0.01

^d^ *p* ≤ 0.05

OR- odds ratio for high nicotine dependence by selected characteristics

95 % CI-95 % confidence interval

Ref. – reference group

### Quitting advice and attempts among highly nicotine dependent daily smokers

The proportion of both heavy and non-heavy nicotine dependent daily smokers who had indicated attempts at quitting during the previous 12 months was similar in all the analyzed countries, with the lowest proportion noticed in Russia (Table 3). Additionally, in Russia, differences between daily smokers highly and not-highly dependent on nicotine who indicated attempts at quitting were statistically significant (24.3 % vs. 27.7 %; *p* < 0.05). Differences between the countries existed regarding the percentages of daily smokers who indicated that health providers had asked if they smoked tobacco, with the highest percentages observed in Romania and the lowest in Russia and Ukraine. In Poland, differences between the heavily and not-heavily dependent smokers who had indicated that health providers had asked them about smoking status were statistically significant (68 % vs. 61 %; *p* < 0.05). Most of the study subjects indicated that their health provider had advised them to quit smoking (with the highest percentages in Romania among the heavily dependent smokers and the lowest percentages in Russia among the not-heavily dependent smokers). In Poland, significantly more heavily dependent smokers had indicated that they were advised to quit smoking by a health provider than the not-heavily dependent smokers (84 % vs. 77 %; *p* < 0.05). Unfortunately, a low proportion of the participants indicated that they were thinking about quitting smoking within the next month, as indicated by 11 % of the not-heavily dependent smokers in Poland and 1.9 % of the heavily dependent smokers in Russia. The percentages are also low for those planning to quit smoking within the next 12 months. A high proportion of the participants had no interest at all in quitting smoking. In addition, a statistically significantly higher proportion of those not thinking about quitting at all were observed among the highly dependent on nicotine smokers compared to the less addicted ones, in all the analyzed countries (51 % vs. 41 %, *p* ≤ 0.001 in Poland, 44 % vs. 33 %, *p* ≤ 0.01 in Romania, 57 % vs. 38 %; *p* ≤ 0.001 in Russia, 44 % vs. 28 %; *p* ≤ 0.001 in Ukraine).

**Table 3:** Quitting advice and attempts among daily smokers

	Poland			Romania			Russia			Ukraine		
	*N* = 1939	Not-heavily dependent smokers	Heavily dependent smokers	*N* = 867	Not-heavily dependent smokers	Heavily dependent smokers	*N* = 3558	Not-heavily dependent smokers	Heavily dependent smokers	*N* = 1865	Not-heavily dependent smokers	Heavily dependent smokers
		*n* = 1405 (72.4 %)	n =534 (27.6 %)		*n* = 647 (74.6 %)	*n* = 220 (25.4 %)		*n* = 2509 (70.5 %)	*n* = 1049 (29.5 %)		*n* = 1382 (74.1 %)	n =483 (25.9 %)
Quit attempts during the past 12 months												
Yes	568	430 (30.6 %)	138 (25.8 %)	301	228 (35.3 %)	73 (33.2 %)	949	694 (27.7 %)	255 (24.3 %)^a^	582	439 (31.8 %)	143 (29.6 %)
No	1371	975 (69.4 %)	396 (74.2 %)	564	417 (64.7 %)	147 (66.8 %)	2605	1812 (72.3 %)	793 (75.7 %)	1282	942 (68.2 %)	340 (70.4 %)
Visited healthcare provider in the last year												
Yes	1171	859 (61.2 %)	312 (58.4 %)	439	341 (52.7 %)	98 (44.5 %)^a^	1810	1300 (36.7 %)	510 (48.9 %)^c^	506	396 (27.1 %)	110 (22.8 %)
No	767	545 (38.8 %)	222 (41.6 %)	428	306 (47.3 %)	122 (55.5 %)	1736	1201 (63.3 %)	535 (51.1 %)	1358	985 (72.9 %)	372 (7725 %)
Healthcare provider asked if smoking tobacco^d^												
Yes	733	521 (60.7 %)	212 (68.2 %)^a^	374	296 (86.8 %)	78 (79.6 %)	864	590 (45.4 %)	274 (53.7 %)^c^	234	179 (45.2 %)	55 (50.0 %)
No	436	337 (39.3 %)	99 (31.8 %)	65	45 (13.2 %)	20 (20.4 %)	946	713 (54.6 %)	236 (46.3 %)	272	217 (54.8 %)	55 (50.0 %)
Healthcare provider advised quitting^e^												
Yes	578	400 (77.1 %)	178 (84.0 %)^a^	318	251 (84.8 %)	67 (85.9 %)	656	442 (75.2 %)	214 (78.4 %)	183	138 (77.1 %)	45 (81.8 %)
No	153	119 (22.9 %)	34 (16.0 %)	56	45 (15.2 %)	11 (14.1 %)	205	146 (24.8 %)	59 (21.6 %)	51	41 (22.9 %)	10 (18.2 %)
Thinking about quiting												
Within the next month	169	138 (11.1 %)	31 (6.4 %)^b^	62	49 (8.0 %)	13 (6.1 %)	85	66 (2.9 %)	19 (1.9 %)	94	70 (5.5 %)	24 (5.2 %)
Within the next 12 month	435	347 (27.9 %)	88 (18.2 %)^c^	137	114 (18.5 %)	23 (10.7 %)^b^	277	219 (9.5 %)	58 (5.9 %)^c^	306	250 (19.6 %)	56 (12.1 %)^c^
Someday but not within the next 12 month	367	251 (20.2 %)	116 (24.0 %)	336	252 (41.0 %)	84 (39.3 %)	1491	1143 (49.6 %)	348 (35.5 %)^c^	784	603 (47.3 %)	181 (39.2 %)^c^
Not interested in quitting	757	509 (40.9 %)	248 (51.3 %)^c^	294	200 (32.5 %)	94 (43.9 %)^b^	1432	878 (38.1 %)	554 (56.6 %)^c^	552	351 (27.6 %)	201 (43.5 %)^c^

^a^ *p* ≤ 0.05 heavily addicted smokers vs not-heavily addicted smokers

^b^ *p* ≤ 0.01 heavily addicted smokers vs not-heavily addicted smokers

^c^ *p* ≤ 0.001 heavily addicted smokers vs not-heavily addicted smokers

^d^restricted to respondents who visited a healthcare provider within the past year

^e^restricted to respondents who visited a healthcare provider within the past year and were asked whether they smoked

## Discussion

Data from the GATS surveys in the four Eastern European countries indicated that more than 25 % of daily smokers can be classified as highly dependent on nicotine based on the Heaviness of Smoking Index. Similar patterns of nicotine dependence were observed in all the analyzed countries. Higher odds of being heavily dependent on nicotine were identified for males, older people and those who had started smoking at a young age. In addition, having fewer restrictions on smoking at home was significantly associated with a high level of nicotine dependence. Awareness of health consequences of both active and passive smoking did not have any significant impact on the level of nicotine dependence in any of the analyzed countries. A high proportion of the daily smokers were not interested in quitting at all, with a significantly higher proportion noticed among the heavily dependent smokers compared to those not-heavily dependent ones.

Our results indicate that the highly dependent on nicotine smokers constitute a quarter of Romanian daily smokers and even more in the remaining three analyzed countries. These results are in agreement with the data from GATS in three South-East Asian countries, where hardcore smokers represent 18–30 % of daily smokers [26]. A slightly higher proportion of hardcore smokers (33 %) was observed in Italy and lower in the US (5.2 % in California, 7.8 % in Missouri) and in England (16 %) [27–30]. Analysis of the prevalence and psychosocial determinants of nicotine dependence in 9 countries of the former Soviet Union has indicated that in the majority of those countries the percentages of people with high nicotine dependence ranged from 20 % to 29 % [31]. The differences in the prevalence of heavy smoking among the studies may result from several factors. Firstly, they can be related to the study design or population. Secondly, different classifications of heavy smoking (based on FTND, HSI or hardcore smoking) can be responsible for the observed differences. This is illustrated by our previous analysis based on the GATS survey in Poland where hardcore smokers constituted 42 % of daily smoking men and 38 % of daily smoking women (compared 32 % and 22 % in the current assessment) [32]. In the previously published analysis hardcore smoking was defined by different variables than those used in HSI in the current assessment, which is the reason for the different results [32]. In addition, the population may differ in terms of their economic, social and cultural context. Finally, tobacco control measures including policy, prevention and intervention activities, can have a high impact on the prevalence of tobacco smoking and the proportion of people heavily dependent on nicotine among the daily smokers.

In agreement with other research, our study shows associations between a younger age at the onset of smoking and HSI [31]. This could be related to psychosocial influence on young people which has been shown to strongly affect adult smoking patterns later in life [31, 33]. The earlier the person starts smoking, the greater the risk of becoming a heavy smoker, dependent on nicotine, and with a lower chance of quitting smoking as an adult. Taking this into account, programs that motivate young people not to start smoking, or that delay the initiation of smoking, might have considerable benefit. Delaying the initiation of smoking among adolescents can result in a reduction in the heavy smoking rate and increase the potential for successful cessation. These activities should focus on education of young people about health consequences of smoking, or creating a fashion for non-smoking among the young, increasing the prices of cigarettes as well as enforcement of a minimum age for buying cigarettes [34].

Findings from GATS in all the four countries indicated that smokers who were heavily dependent on nicotine were more likely to live at homes where smoking is allowed, or where there are no rules regulating smoking at home, than the not-heavily dependent smokers. This is in agreement with other studies showing that smoke-free homes are associated with an increased cessation of smoking and reduced smoking frequencies in adult smokers [35, 36]. Creating a smoke-free environment could be one of the strategies which, in the wider perspective, could result in reducing the number of daily smokers, and among them heavy smokers.

In our analysis, we observed a higher risk of high nicotine dependence among people with a primary educational level compared to those with a high level of education (in Poland and Ukraine) and among the unemployed comparing to the employed population in Ukraine. This has been also proven in other studies showing that the less educated, unemployed and low-income smokers are more intensely dependent on nicotine, and are likely to require more support to stop smoking [31, 37]. Those studies underscore the importance of policy measures as well as prevention and cessation interventions for smokers highly dependent on nicotine, which need to take into account the social gradient in smoking patterns. The target group for such activities should cover people from poorer socio-economic backgrounds. Intervention should include price increases for tobacco products, which has been shown to be the best intervention in reducing inequalities in smoking, as disadvantaged smokers were relatively more likely to respond to price increases [38]. Based on the existing estimates, a 70 % increase in the price of tobacco could prevent up to a quarter of all smoking-related deaths worldwide [34].

For the people who are addicted to nicotine, especially those with a lower socio-economic status, some forms of low or no cost help in quitting smoking are crucial. The health-care systems in countries hold the primary responsibility for treating tobacco dependence, with a variety of methods covering: smoking cessation advice incorporated into primary health-care services, and access to low-cost pharmacological therapy. Existing evidence indicates that advice from doctors, structured interventions from nurses and individual and group counselling are effective in helping people to quit smoking [39]. The guidelines to clinicians and health care delivery systems indicate that it is essential to consistently identify and document tobacco use status and treat every tobacco user seen in a health care setting [40]. In addition, taking into account that tobacco dependence treatments are both clinically effective and highly-cost effective, compared to the interventions for other clinical disorders, they should be broadly available and utilized. According to the current recommendations, all the patients who smoke ≥ 10 cigarettes per day should use pharmacotherapy at every quit attempt, unless contraindicated [41]. Nicotine replacement therapy and bupropion are modestly effective, as they help some 5 % to 15 % of the users to remain long term abstinent from smoking, depending on the product and context [42]. A new medication, varenicline, has been found to be more effective than placebo (pooled risk ratio (RR) from meta-analysis = 2.3) and bupropion (pooled RR = 1.5) [43]. It need to be pointed that new treatments that are more effective, safer, cheaper and that can be more widely applied are still needed. Treatment should be offered as one of the components of comprehensive tobacco-control programs that include restrictions on smoking in public places, increased taxes on tobacco, education emphasizing the dangers of tobacco and benefits of cessation, as well as restrictions on tobacco-product marketing. These are critical for cessation efforts, treatment utilization and the maintenance of tobacco abstinence [44]. Since the people with a heavy smoking index have knowledge of smoking hazards but are unwilling to quit, an individualized approach elaborating on the health risks and some motivational methods may be tried [26]. As it is shown in our study, differences between the countries regarding such practices exist. Health-care providers asked 80 % of the heavily dependent smokers in Romania and only 45 % in Russia and Ukraine, if they smoked tobacco. Additionally to this, a high proportion of the study participants in all the analyzed countries were not interested in quitting smoking. This indicates that the crucial role of a health-care provider including primary health care is not fully or properly utilized as a means of motivating and helping people to quit smoking. The low availability of tobacco cessation services in these countries is mainly caused by the weaknesses of the health systems.

The current analysis has several strengths. The Global Adult Tobacco Survey (GATS) is a cross-sectional nationally representative survey and covers a large number of respondents obtained from a general population framework, assuring the reliability and validity of the results. In addition, the results presented by this study are based on the HSI, which is a frequent and reliable measure of nicotine dependence. Moreover, it considers a number of various potential predictors of nicotine dependence.

Limitations of the study also need to be pointed. Firstly, different response rates were observed in the analyzed countries. Differences in the response rates may result from the country’s socio-cultural norms, the level of trust and acceptance of being interviewed on sensitive issues However, the differences are only related to the general response rate and do not generate selection bias in the study. Secondly, for the purpose of this paper, we selected the subjects who were 26 years or older at the time of the survey, because younger people might have still been engaged in the process of initiation of smoking [45]. Moreover, the subjects under 26 might not have completed the maximum level of education [46], making this group unreliable.

## Conclusions

Data from the GATS surveys in four Eastern European countries indicated that smokers highly dependent on nicotine constitute a quarter of the Romanian daily smokers and even more in the remaining countries. Similar patterns of nicotine dependence were observed in all of the investigated countries showing that male gender, younger age at the smoking onset, and fewer restrictions on smoking at home were significantly associated with higher nicotine dependence. The study also highlighted the fact that a high proportion of the participants had no interest in quitting smoking. Although the results relating to the impact of educational and occupational level on high nicotine dependence are not consistent across all the analyzed countries (with the increased risk among the people with primary and vocational levels of education in Poland, primary school in Ukraine, secondary level of education in Russia and Ukraine and unemployed people in Ukraine) they generally indicated existing of social gradient in tobacco dependence. These results underscore importance of policy measures as well as prevention and cessation interventions for smokers who are highly dependent on nicotine. While designing services for heavy smokers to quit, more emphasis should be put on the treatment of nicotine dependence, as well as on the inclusion of behavioral approaches into motivational methods. Along with an individual approach to the treatment of tobacco dependence, a supportive environment is needed to encourage smokers in their attempts to quit.
